# Studying the Binding Modes of Novel 2-Aminopyridine Derivatives as Effective and Selective c-Met Kinase Type 1 Inhibitors Using Molecular Modeling Approaches

**DOI:** 10.3390/molecules26010052

**Published:** 2020-12-24

**Authors:** Qianwen Ye, Chenggong Fu, Jiazhong Li

**Affiliations:** School of Pharmacy, Lanzhou University, 199 West Donggang Rd., Lanzhou 730013, China; yeqw18@lzu.edu.cn (Q.Y.); fuchenggong@lzu.edu.cn (C.F.)

**Keywords:** c-Met kinase, 2-Aminopyridine, 3D-QSAR, molecular docking, molecular dynamics simulations

## Abstract

The mesenchymal epithelial cell transforming factor c-Met, encoded by c-Met proto-oncogene and known as a high-affinity receptor for Hepatocyte Growth Factor (HGF), is one of the receptor tyrosine kinases (RTKs) members. The HGF/c-Met signaling pathway has close correlation with tumor growth, invasion and metastasis. Thus, c-Met kinase has emerged as a prominent therapeutic target for cancer drug discovery. Recently a series of novel 2-aminopyridine derivatives targeting c-Met kinase with high biological activity were reported. In this study, 3D quantitative structure-activity relationship (QSAR), molecular docking and molecular dynamics simulations (MD) were employed to research the binding modes of these inhibitors.The results show that both the atom-based and docking-based CoMFA (Q^2^ = 0.596, R^2^ = 0.950 in atom-based model and Q^2^ = 0.563, R^2^ = 0.985 in docking-based model) and CoMSIA (Q^2^ = 0.646, R^2^ = 0.931 in atom-based model and Q^2^ = 0.568, R^2^ = 0.983 in docking-based model) models own satisfactory performance with good reliabilities and powerful external predictabilities. Molecular docking study suggests that Tyr1230 and Arg1208 might be the key residues, and electrostatic and hydrogen bond interactions were shown to be vital to the activity, concordance with QSAR analysis. Then MD simulation was performed to further explore the binding mode of the most potent inhibitor. The obtained results provide important references for further rational design of c-Met Kinase type I inhibitors.

## 1. Introduction

Cancer is one of the severe life-threatening diseases [1]. Existing data indicated that more than 50% of proto-oncogenes and oncogene products are tyrosine kinases [2]. It was reported that many human tumors were related with abnormal activation of tyrosine kinase route [3,4]. Cellular mesenchymal-epithelial transition factor (c-Met), known as a high affinity receptor for hepatocyte growth factor (HGF), is a member of receptor tyrosine kinases (RTKs). The HGF/c-Met signaling pathway can regulate various tumor cellular processes including growth, invasion and metastasis. Thus, c-Met kinase has emerged as a prominent therapeutic target for cancer drug discovery. Looking for novel and efficient c-Met inhibitors has become a hotspot in the pharmaceutical industry [5].

According to the different structures and binding modes to the c-Met kinase, the reported c-Met inhibitors can mainly be divided into three types. Type I c-Met inhibitors are believed to be ATP-competitive inhibitors that bind to the ATP binding site in a U-shaped conformation, such as Crizotinib 1 [6], S49076 2 [7], AMG 337 3 [8] and Volitinib 4 [9]. Type I c-Met inhibitors are believed to be highly selective for c-Met. Type II c-Met inhibitors, characterized by relatively high molecular weight and lipophilicity [10], such as Cabozantinib 5 [11], Foretinib 6 [12], Altiratinib 8 [13] and Merestinib 9 [14], are also ATP-competitive compounds. But they can pass the gatekeeper and occupy the deep hydrophobic back pocket, and they are multi-target c-Met kinase inhibitors. Type III c-Met inhibitors are other atypical c-Met kinase inhibitors such as Tivantinib 10 (ARQ197) [15]. Type III c-Met inhibitors are identified as non-ATP competitive inhibitors that binds to an inactive c-Met conformation.

Some potential type I c-Met inhibitors, such as 2-aminopyridine derivatives have an important nitrogen heterocyclic structure skeleton with a wide range of biological activities and excellent activities against tumors, which makes it a potential c-Met RTK inhibitors for cancer therapy [16].

In this study, in order to explore the quantitative structure-activity relationship (QSAR) [17] of these new 2-aminopyridine c-Met kinase type I inhibitors. 3D comparative molecular field analysis (CoMFA) [18] and comparative molecular similarity indices analysis (CoMSIA) [19] methods were carried out to construct QSAR models to reveal the key structural features related to their inhibition activity. Two different methods were used to obtain the optimal molecular conformation alignments, one is based on atomic alignment (Alignment A) and the other is based on molecular docking conformation (Alignment B). Then we used molecular dynamics simulations (MD) method to study the binding mode of the most active compound and C-Met. The obtained results can help to understanding the inhibition mode and provide useful guidance for the rational design of novel c-Met inhibitor with higher activities.

## 2. Results and Discussion

### 2.1. The Studied c-Met Inhibitors

The studied dataset is composed of 42 novel 2-aminopyridine derivatives [20,21]. These compounds were designed by way of replacing the imidazole, oxazole or tetrazole at the C-3 position with the amide bond and introducing a suitable aryl group on the amide bond or replacing the known O-linker with amide, sulfonamide and S-linkers [20,21].Their inhibitory activities were obtained from the literatures published by the same laboratory [20,21]. These compounds were divided into a training set (34 compounds used to build model) and a prediction set (8 compounds used to validate the constructed model, marked by ‘*’ in Table 1) in an approximate ratio of 4:1. For QSAR analysis, the half maximal inhibitory concentration (IC_50_) values, inhibitory activities on c-Met kinase of 2-aminopyridine derivatives, were transformed into pIC_50_ values (−log IC_50_) and used as dependent variables (Supplementary Materials).

### 2.2. Atom-Based CoMFA and CoMSIA Models

#### 2.2.1. The 3D-QSAR Models

**Figure 1:**
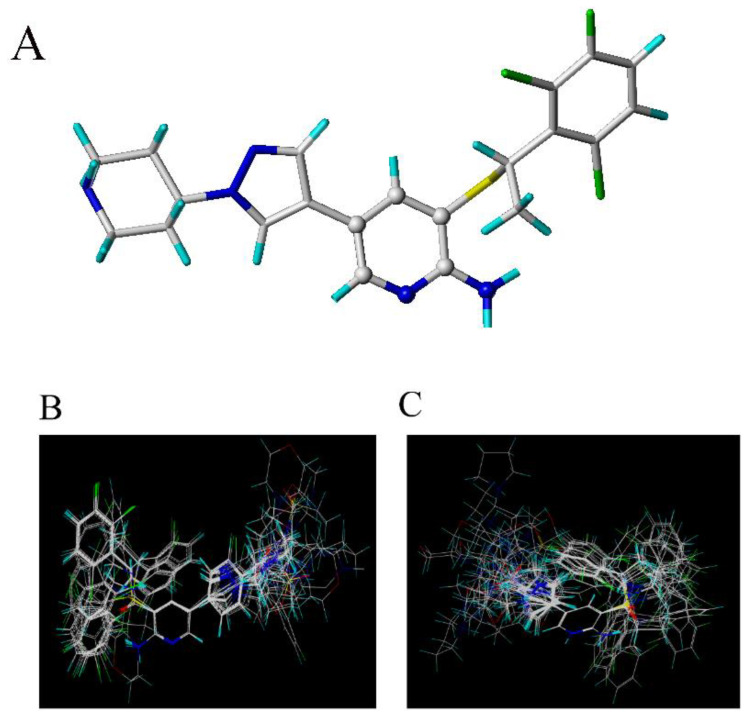
The studied c-Met inhibitors. (**A**) is the common atoms (represented as balls) used for molecular alignment in compound **4**. (**B**) is the atom-based alignment figure (Alignment A). (**C**) is the docking-based alignment figure (Alignment B).

To investigate the relationships between molecular structures and corresponding activities, 42 potential c-Met Kinase inhibitors were firstly analyzed by CoMFA and CoMSIA methods. Figure 1 shows the overlay of all training compounds using the common atoms of molecule 4 as a template (Alignment A). The LOO cross-validation correlation coefficient Q^2^, correlation coefficient R^2^, standard error of estimate (SEE) and F-statistic value were used to evaluate the reliability of the model. The corresponding values of the parameters were listed in Table 2.

#### 2.2.2. CoMFA Results

**Figure 2:**
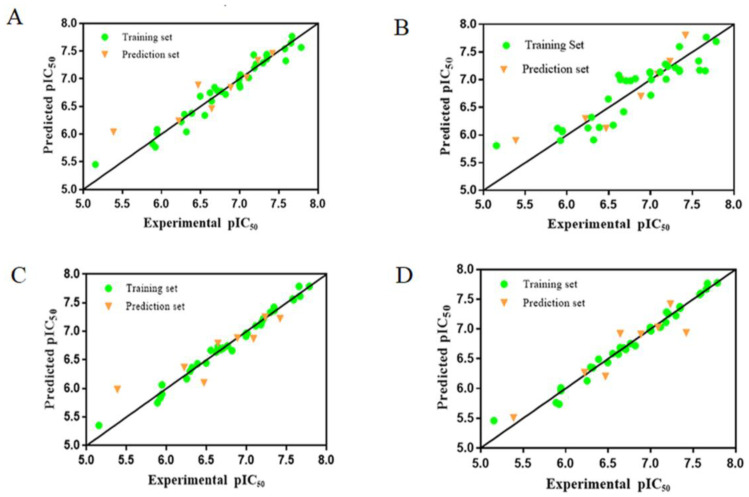
The scatter plots of experimental values vs. predicted values using CoMFA and CoMSIA based on atom-based approach (Alignment A) and docking-based approach (Alignment B). (**A**) is the CoMFA model of Alignment A, (**B**) is the CoMSIA model of Alignment A, (**C**) is the CoMFA model of Alignment B, (**D**) is the CoMSIA model of Alignment B.

**Figure 3:**
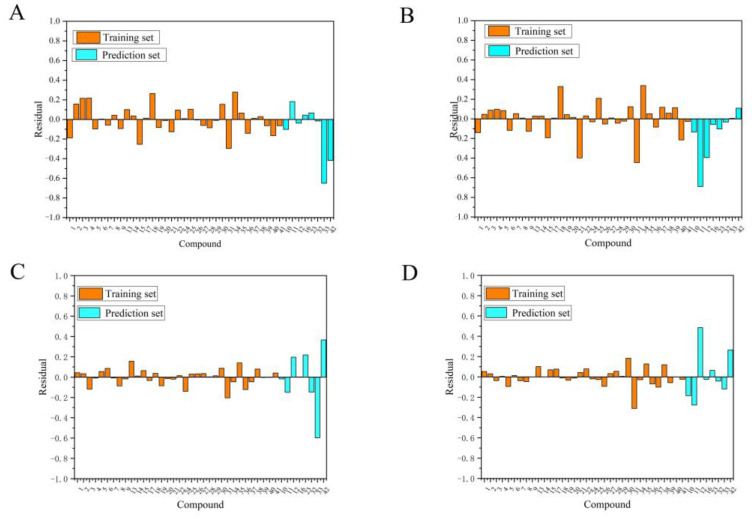
The errors between the experimental values and the predicted values for CoMFA and CoMSIA based on ligand-based approach and receptor-based approach were shown in panels (**A**–**D**), respectively. The shorter the bar is, the better the model prediction is.

An ideal QSAR model should be stable, reliable and highly predictive. It is generally considered that Q^2^ and R^2^ (Q^2^ > 0.5, R^2^ > 0.6) are the basic parameters to evaluate a 3D CoMFA model [22]. The parameter values listed in Table 2 indicated that the built CoMFA model has good stability and robustness. Figure 2A shows the scatter plot of experimental and predicted activity values. In this figure, the samples are evenly distributed around the straight line y = x. All values are within the acceptable range. Figure 3A shows the error between the experimental and the predicted activity values, in which 20 compounds have positive errors and 22 compounds have negative errors.

#### 2.2.3. CoMSIA Results

In the CoMSIA analysis, in addition to the electrostatic and steric fields, hydrophobic field and hydrogen bond field (including hydrogen bond acceptor and hydrogen bond donor) are also used. A total of eight different field combinations were calculated to find optimal model. The results and parameters were listed in Table 3. From this table we can see that the model by using S, E, A and D fields can provide the highest Q^2^ with contributions of 19.7%, 33.2%, 19.3% and 27.8%, respectively. According to the LOO results, the optimal principal component number is 2 and R^2^ is higher than 0.9. The scatter plot of the experimental and predicted activity values of the CoMSIA model was shown in Figure 2B. Figure 3B is the predicted error of each chemical in a bar graph, in which 23 compounds showed a positive error and 19 compounds showed a negative error. All these results indicated that the CoMSIA model is also robust and stable with high predictive ability.

#### 2.2.4. Contour Map Analysis

The contour maps can intuitively show the relationships between biological activities and structures, and provide useful guidance for the rational design of new inhibitors.

**Figure 4:**
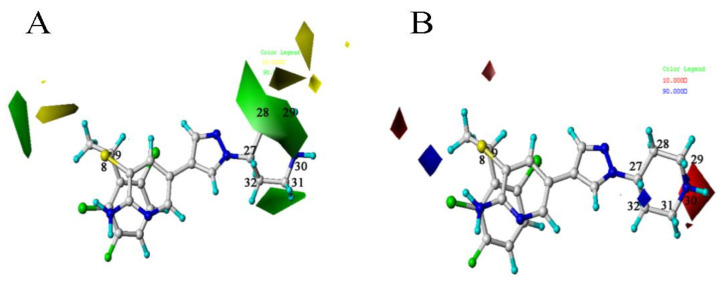
The CoMFA STDEV × COEFF contour maps based on template compound **4**. (**A**) The steric field. (**B**) The electrostatic field.

The CoMFA contour maps were shown in Figure 4. The green area in Figure 4A indicates that the introduction of a large group at this position will increase the activity of the c-Met inhibitor. The yellow area indicates that the introduction of a large group can decrease the inhibition activity. There is a green area around the C28 atom of the template molecule. Compounds **4** and **6** are chosen as comparison to prove this point for the reason that 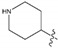 is larger than –CH_3_, so the activity of compound **4** is higher than compound **6**. There is a yellow area near the C8 position. Taking compounds **4** and **1** as well as compounds **5** and **2** as examples, –S at the C8 position is smaller than 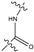, so the corresponding compounds have higher activity values.

In the electrostatic contour map, the red areas indicate that the electronegative groups can increase the activity of the compound. In Figure 4B, there is a red area near C30 position. The compounds **2** and **1** can be the proof of this point. The activities of compounds with group -O are higher than those with the -N near C30. The blue area near C32 indicates that the introduction of an electropositive group at this position is beneficial to the activity, for example, the bioactivity of compound **9** with 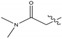 is higher than compound **8** with 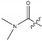.

**Figure 5:**
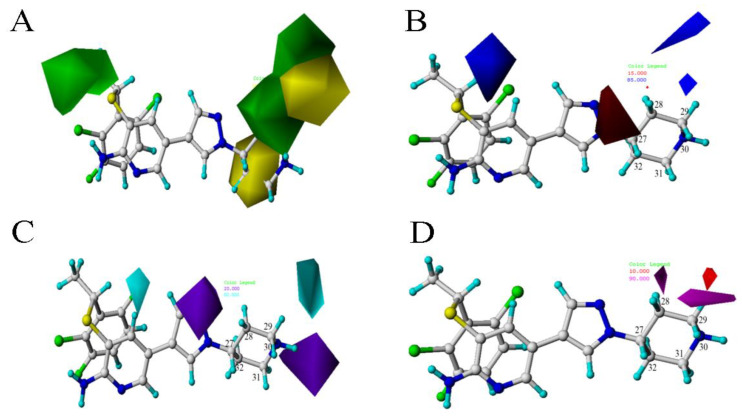
The CoMSIA STDEV × COEFF contour maps matched with template compound **4** based on Alignment A. (**A**) The steric field. (**B**) The electrostatic field. (**C**) The hydrogen bond donor field. (**D**) The hydrogen bond acceptor field.

The contour maps of CoMSIA were shown in Figure 5, from where we can see that the steric and electrostatic figures are mainly similar to the CoMFA model. Figure 5C is the hydrogen bond donor field. The cyan and purple regions indicate that the H-bond donor is favorable or unfavorable to the activity. The purple area near C30 position can be proved that the activity of compound 2 with -O is higher than compound **1** with the -N. Figure 5D is the contour map of the hydrogen acceptor field. The magenta and red regions indicate that the H-bond acceptor is favorable or unfavorable to the activity. The hydrogen acceptor groups near the C29 position can increase the biological activity of the c-Met inhibitor, which can be demonstrated by the following comparison: compounds **4** and **5**, replacing 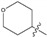 with 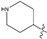 group at this position can increase the activity.

### 2.3. Docking-Based CoMFA and CoMSIA Models

#### 2.3.1. Molecular Docking Analysis

In order to find reasonable inhibitor active conformation, rather than the lowest energy conformation, we conducted another two QSAR models using the docked conformations. Here, CDOCKER module in the Discovery Studio 2.5 [23] was used to complete the molecular docking and The X-ray crystal structure of c-Met kinase (PDB ID:3CCN) was downloaded from the RCSB protein database (http://www.pdb.org). In order to test the accuracy of the docking method, we docked the small molecule contained in the X-ray crystal, and compared the best conformation with the configuration of the small molecule in the X-ray crystal. The results showed that the root mean square deviation (RMSD) between the optimal docking conformation of the compound and its X-ray crystal conformation was 1.0294 Å, which was lower than 2 Å, the value described in the literature reference. This result suggests that the docking method can accurately predict the configuration and orientation of the compound reported in the X-ray crystal structure, and the docking method is reliable and can be used for c-Met kinase inhibitor docking.

**Figure 6:**
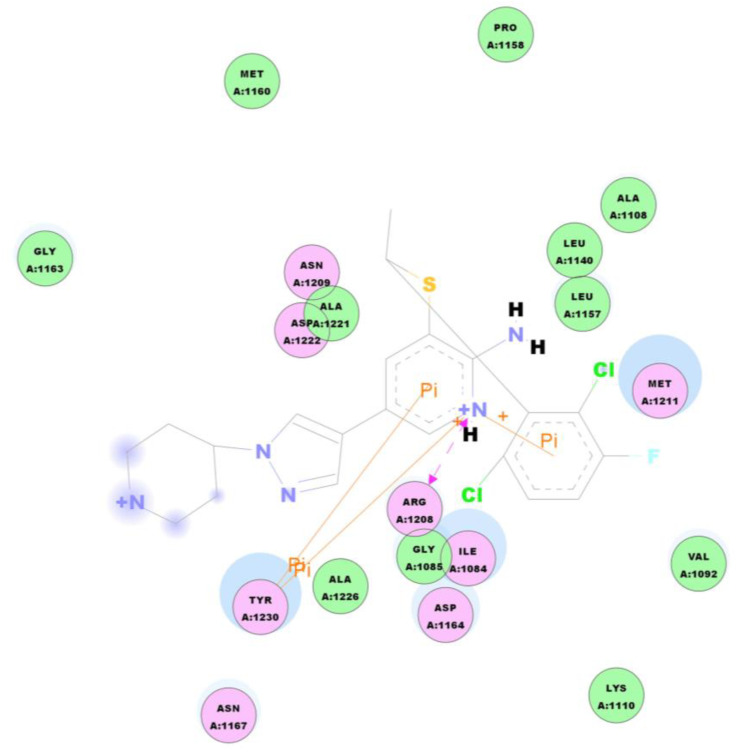
2D diagram of docking interaction of compound **4** with c-Met Kinase 3CCN.

After conformation minimization, the forty-two compounds were docked into the active pocket of c-Met kinase 3CCN, and 3129 binding conformations were obtained, where the CDOCKER score ranged between −16.84 and 980.98 Kcal/mol. The most active compound **4** was chosen as an example to analyze the interaction mode with c-Met Kinase, shown in Figure 6. From this figure it can be seen that the hydrogen bond and Pi are two most important interactions.

#### 2.3.2. Docking-Based CoMFA and CoMSIA Model Results

After molecular docking, the best-scoring conformation of each compound was selected for the subsequent QSAR analysis. The training set and prediction set used here are the same as the atom-based QSAR analysis. Table 4 displays the results of different field combinations in the CoMSIA model. It can be seen that the four combined fields of SEAD is the best one, the same as the atom-based model. The statistical parameters of these two models were shown in Table 2. The correlation diagrams of experimental and predicted values of CoMFA and CoMSIA were shown in Figure 2C,D, respectively. The error bar graphs between the experimental and predicted values were shown in Figure 3C,D, respectively.

The alignment graph based on the docking conformation is shown in Figure 1C. Compared with atom-based graph, the docking-based alignment graph is slightly dispersed, and the model Q^2^ is a little lower. But the docking-based CoMFA and CoMSIA models are still satisfactory and practically useful, for the Q^2^ value higher than 0.50, and other parameters are all similar or even higher than the atom-based models.

#### 2.3.3. Contour Map Analysis

**Figure 7:**
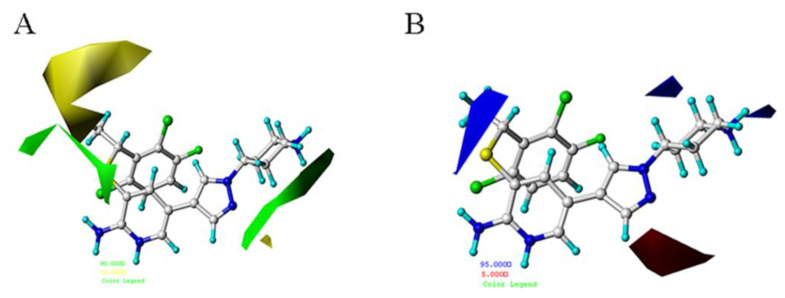
The docking-based CoMFA STDEVCOEFF contour maps (**A**) The steric field. (**B**) The electrostatic field.

**Figure 8:**
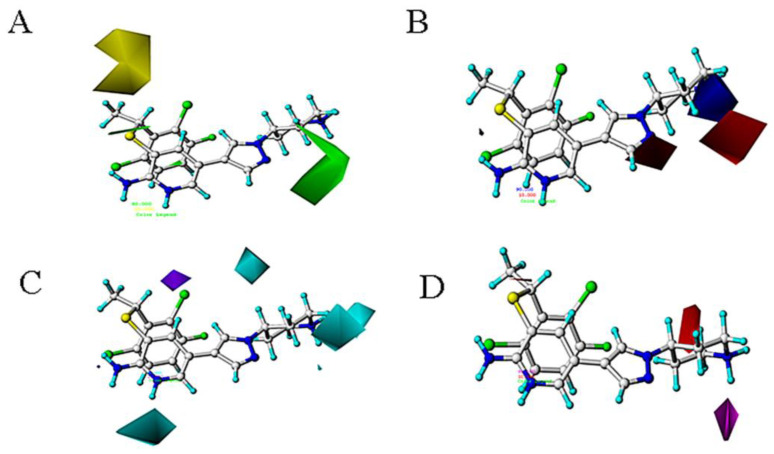
The docking-based CoMSIA STDEV × COEFF contour maps (**A**) The steric field. (**B**) The electrostatic field. (**C**) The hydrogen bond donor field. (**D**) The hydrogen bond acceptor field.

A substantial amount of information contained in the CoMFA and CoMSIA models can be seen through the contour map. Figure 7 and Figure 8 respectively show the contour maps of docking-based CoMFA and CoMSIA models. Comparing these figures with those from atom-based results, it can be seen that, though slightly complicated and different, they provide quite similar relationships between structure and activities.

### 2.4. Molecular Dynamics Simulation Results

**Figure 9:**
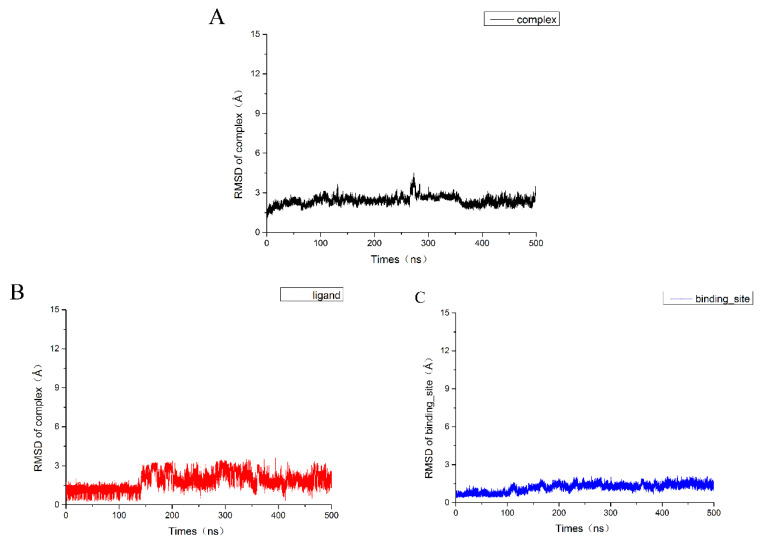
Root mean square deviation (RMSD) of the system. (**A**) the RMSD of the protein backbone atoms of the complex; (**B**) the RMSD of the heavy atoms (all non-hydrogen atoms) of the ligand; (**C**) the RMSD of the Cα atoms the binding site that the residues within 5 Å around the ligand.

In order to further study the interaction mode between these inhibitors and c-Met, molecular dynamics simulation was carried out on the system of the most active compound **4** bound to 3CCN via molecular docking. We ran a 500 ns molecular dynamics simulation and analyzed the trajectory. The root mean square deviation (RMSD) and the root mean square fluctuation (RMSF) of the complex, the ligand and the binding pocket (defined as residues within 5 Å around the ligand) were calculated to research the stability of the structure and residues in the system. As shown in Figure 9A, there were some fluctuations in the entire system at the early stage of the trajectory and the fluctuations of the RMSD in the complex indicating the system was gradually stabilized. In order to obtain more accurate analytical results, we chose the last 50 ns trajectory for the next analysis.

**Figure 10:**
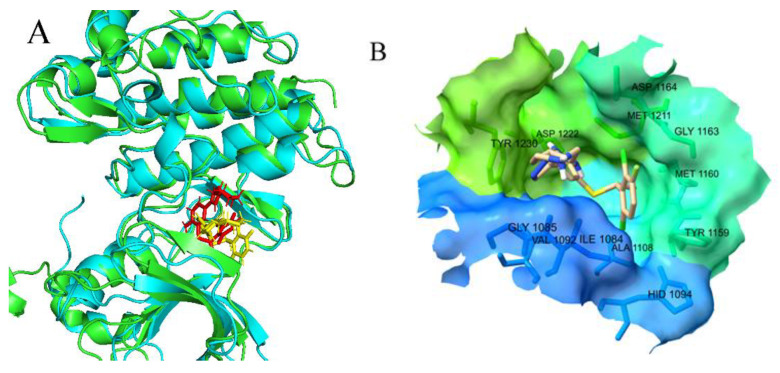
The ligand location comparison of the initial structure and after MD stabilized conformation. (**A**) Overlay of the first frame structure and the last frame structure of the trajectory, this green is the protein structure of the first frame and this red is the small molecule structure of the first frame, this blue is the protein structure of the last frame and this yellow is the small molecule structure of the last frame; (**B**) the amino acid residues around the binding site.

Then, we extracted the structure of the last frame and compared it with the initial structure to determine whether the molecule was stable in the binding site. As shown in Figure 10A, though the conformation of compound **4** had undergone some changes, it still remained locating in the allosteric pocket.

**Figure 11:**
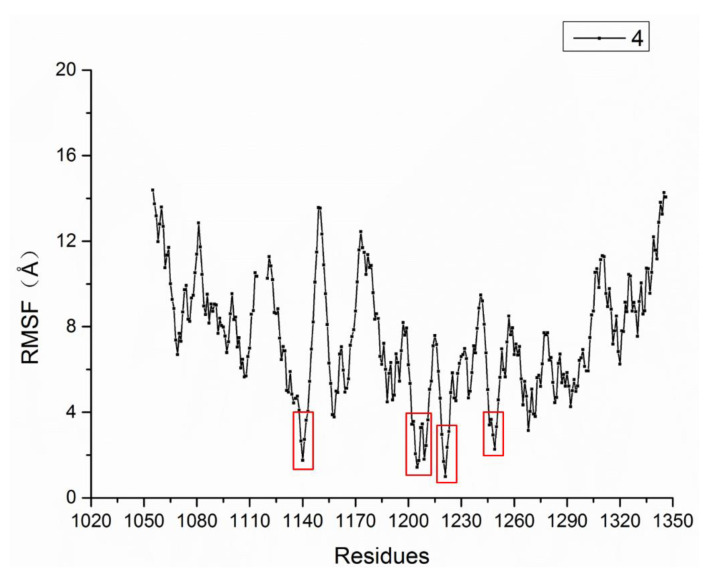
Root mean square fluctuation (RMSF) of the system.

We calculated the RMSF of every Cα atom of the complex. As shown in Figure 11, the RMSF values of the residues in the region of 1139–1142, 1200–1213, 1219–1225 and 1245–1250 were smaller than other residues. These residues, such as ASP1222 and TYR1230 that were shown in Figure 10B, were near to the c-Met binding site, which illustrated that the residues near the binding site were more stable than other residues during the MD process. This result is consistent with the result of the RMSD of the binding site in Figure 9C.

The binding energy between the ligand and protein was calculated by using MM-GBSA method, listed in Table 5. From the value of binding free energy (∆G_bind_), we can conclude that Compound **4** bind well to the protein. As shown in Table 5, van der Waals (∆E_vdW_) contributes the most to the binding of the ligand and protein. The contribution ratio of ∆E_vdW_ and ∆E_ele_ to the system was similar to that in the CoMFA model. 

**Figure 12:**
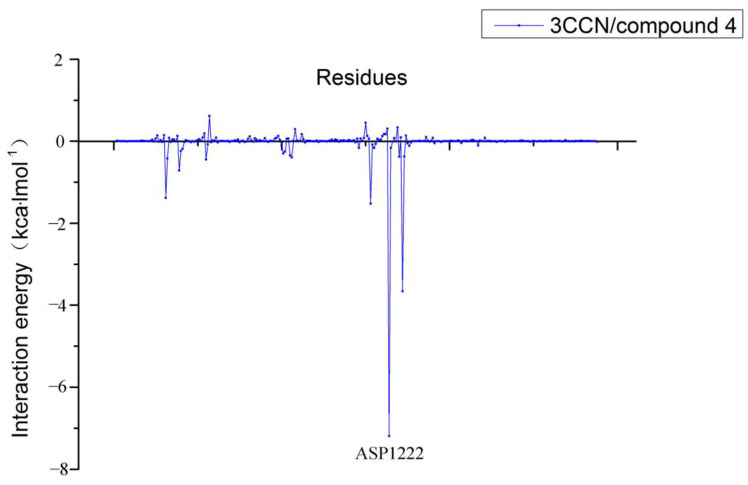
Contribution of partial residues calculated by decomposing the binding energy.

In addition, we decomposed the binding energy to study the contribution of each residue, shown in Figure 12. In this figure, the residue ASP1222 has lowest energy value, indicating that ASP1222 has the greatest contribution to the binding of the system.

In order to clarify the reason why residue ASP1222 is so important, we calculated the hydrogen bonds, defined as the distance of acceptor and donor < 0.35 nm and the angle > 120° [24], of the entire system during the MD simulation. As shown in Table 6, we can clearly see that the main hydrogen bonds of the whole system were formed only by the residue ASP1222 and Compound **4**.

## 3. Materials and Methods

### 3.1. Molecular Conformation

The structures of the studied forty-two compounds were drawn and optimized in SYBYL 6.9 software [25]. Gasteiger-Hückel charge of each compound is calculated [26]. To obtain the lowest energy conformation of each molecule, a conformation searching was carried out through the multiple reconstruction image search method module. Then the lowest-energy geometric structure of each molecule was selected for the succeeding molecular alignment.

### 3.2. Molecular Docking

In this study, the CDOCKER module is used to do molecular docking in the Discovery Studio 2.5 [23] to generate the molecular active conformations of the c-Met kinase inhibitors. The molecular structures were optimized by preparing binding ligand modules. The X-ray crystal structure of c-Met kinase, binding with triazolopyridazine, is downloaded from the RCSB protein database (http://www.pdb.org) and the PDB succession number is 3CCN. The protein structure was prepared using the protein preparation module, including adding missing loop structure regions, protein protonation and automatic protein preparation. The torsion force of the ligand and the receptor were set to be rotatable and rigid respectively, by using the semi-flexible docking method [27]. The binding site radius was set to 10, top hits were set to 100, and other parameters were set as default. According to the scoring and binding mode of each molecule, the best conformation was selected for subsequent analysis.

### 3.3. Molecular Alignment

To obtain optimal QSAR models, two different alignment methods, namely alignment A and alignment B, were employed. Alignment A is the atom-based method. The compound with highest inhibition activity is selected as the template molecule, and the remaining compounds were aligned to the common substructure using “align database” command. Alignment-B is a docking-based approach, all the conformations used for alignment were derived from the docking analysis, i.e., the best conformation of each molecular docking was selected, then imported into SYBYL6.9, added the Gasteiger-Hückel charges and subjected to the process of molecular alignment. 

### 3.4. CoMFA and CoMSIA Modeling

The entire process of 3D QSAR research was completed in SYBYL 6.9 software. In this study, the lowest energy conformation database of the training set compounds were obtained as stated in Section 3.1. The most active compound No 4 was chosen as the template molecule, and the common framework superposition method was used to ensure the consistency of the spatial orientation as much as possible.

CoMFA analysis calculated steric and electrostatic fields as input by applying Tripos force field. Methane molecule and hydrogen ion were used as probes to travel in the space around the molecule to detect corresponding steric field (S) and electrostatic field (E) values. The cutoff value and attenuation factor were set default to 30 kJ/mol and 0.3 [28], respectively.

On the bases of steric and electrostatic fields, CoMSIA method also uses another three fields, i.e., hydrogen bond acceptor (A), hydrogen bond donor (D) and hydrophobic field (H). Gaussian function, a function related to distance, was used to calculate these field values.After model building, the Stdev*Coeff method was adopted to display the contour maps of the model results.

### 3.5. Model Validation

Leave-one-out (LOO) cross-validation is employed to determine the optimal principal component number (ONC), and partial least squares (PLS) was carried out to build models [24]. LOO cross-validation is the method that extracts a compound from the training set as a temporary test set, and uses the remaining compounds as a temporary training set to build a model. Use the model from the temporary training set to predict a compound in the temporary test set. PLS is an extension of multiple regression analysis that we used pIC_50_ value as the dependent variable and the descriptors of CoMFA or CoMSIA as the independent variable in this study The parameters used in the model building process include the optimum number of components (ONC), cross-validation correlation coefficient (Q^2^), standard error of estimate (SEE), correlation coefficient (R^2^) and F-statistic value (F).The LOO cross-validated coefficient Q^2^ value is calculated as follows (Equation (1)):

(1) $$Q^{2}\;=\;1-\frac{\sum_{i\;=\;1}^{n}{\left(Y_{\mathrm{pred},i}-Y_{\exp,i}\right)}^{2}}{\sum_{i\;=\;1}^{n}{\left(Y_{\exp,i}-Y_{\mathrm{mean}}\right)}^{2}}$$

In this formula, Y_exp_ and Y_pred_ represent the experimental activity and the predicted activity, respectively. Y_mean_ is the average activity of all molecules in the training set.

The predictive ability of the models was assessed by the $R_{\mathrm{pred}}^{2}$, defined as follows (Equation (2)):

(2) $$R_{\mathrm{pred}}^{2}=\frac{\mathrm{SD}-\mathrm{PRESS}}{\mathrm{SD}}$$

where SD is the sum of the squared deviations of the actual activity of the molecule in the prediction set and the average activity of all molecules in the training set. PRESS indicates the sum of squared deviations of the experimental and predictive biological activity values of the prediction set compounds.

### 3.6. Molecular Dynamics Simulation

In order to perform molecular dynamics simulations, we used the restrained electrostatic potential (RESP) protocol with HF/6-31G* basis set to calculate the partial atomic charges for the ligand atoms [29,30,31]. The force field parameters for the ligand were created using the Antechamber program and described by the General Amber Force Field (GAFF) [32]. The force filed parameters for the protein was generated by a standard ff14SB [33] force field. The system was built with the tLEaP module of the Amber 14 package [34]. Then, we used a cubic box of TIP3P [35] water molecule to wrap the system and each amino acid in the system was at least 10 Å from the edge of the water box. The chloride ions were added to the system to make it in an electrically neutral state. In this part, AMBER 14 will renumber the amino acid sequence, so the number of the amino acid of protein will change from aa 1055–1114 and aa 1120–1346 to aa 1–60 and 61–287.

All molecular dynamics simulations were carried out using the AMBER 14 package. The process of energy minimization was divided into three steps. First, the system was constrained by 5.0 kcal·mol^−1^·Å^−2^ to optimize solvent and ionic molecules. Then, only the protein backbone atoms were constrained by 3.0 kcal·mol^−1^·Å^−2^ to make amino acid side chains find better ways to accommodate the ligand. In the third step, no restriction was imposed on the atoms to minimize the system. In every step, we executed 5000 steps with the first 2500 steps executed by using the steepest gradient descent method and the second 2500 steps performed by using the conjugate gradient method. We heated the system from 0 K to 310 K in the canonical (NVT) ensemble after energy minimization and this process lasted 100 ps when all atoms were constrained by 5.0 kcal·mol^−1^·Å^−2^. To adjust the solvent density to equilibrate, a short equilibration simulation over 50 ps under 1 atm pressure in the isothermal isobaric (NPT) ensemble was performed and all atoms of the system was restrained by 5.0 kcal·mol^−1^·Å^−2^. After this, a trajectory of 1.5 ns was performed in NPT ensemble. The first 1.0 ns was divided into five steps and every step lasted 0.2 ns, and the limiting force for the five steps were set as 5.0, 4.0, 3.0, 2.0 and 1.0 kcal·mol^−1^·Å^−2^, respectively. The last 0.5 ns were carried out without any restraint.

We used the PMEMD program to perform a 500 ns production of MD simulation of the system at 310.0 K, 1 atm in the NPT ensemble without any restraint. During the simulation, we used the Particle Mesh Ewald (PME) [36] method to deal with long-range Coulomb interactions and the SHAKE algorithm [37] to limit the bond length containing hydrogen atoms. The cutoff value was set to 10.0 Å to handle non-bonded interactions. In order to avoid fringe effect, periodic boundary conditions was used during simulation. We set 2 fs as the time step and recorded the coordinates of the track every 2 ps in this process.

### 3.7. Binding Free Energy Calculations

MM-GBSA [38,39,40,41] method was used to calculate the binding free energy of the system. In this process, an average of 5000 structures was extracted with an interval of 10 ps from the last 50 ns MD trajectory. We used the equations below to calculate the binding free energy (Equation (3)):

(3) $$\Delta G_{\mathrm{bind}}\;=\;\Delta G_{\mathrm{complex}}-\Delta G_{\mathrm{receptor}}-\Delta G_{\mathrm{ligand}}\;\Delta G_{\mathrm{bind}}\;=\;\Delta H-T\Delta S\approx \Delta E_{\mathrm{MM}}+\Delta G_{\mathrm{solv}}-T\Delta S\;\Delta E_{\mathrm{MM}}\;=\;\Delta E_{\mathrm{internal}}+\Delta E_{\mathrm{ele}}+\Delta E_{\mathrm{vdW}}\;\Delta G_{\mathrm{solv}}\;=\;\Delta G_{\mathrm{GB}}+\Delta G_{\mathrm{NP}}$$

The binding free energy (∆G_bind_) is the sum of the enthalpy term (∆H) and entropy term (−T∆S). ∆H of the system is the summation of the interaction energy of the gas phase among the protein–ligand (∆E_MM_) and the solvated free energy (∆G_solv_). ∆E_MM_ is obtained by adding the internal energy (∆E_internal_, consists of the energies of bonds, angels and torsions), the electrostatic interaction energy (∆E_ele_) and the van der Waals interaction energy (∆E_vdW_). ∆G_solv_ is the sum of the polar solvation free energy (∆G_GB_) and the nonpolar solvation free energy (∆G_NP_).

### 3.8. Per-Residue Free Energy Decomposition Analysis

We decomposed per-residue free energy decomposition using the 5000 structures collected from the last 50 ns MD trajectory with an interval of 10 ps. The MM-GBSA method was employed to calculate the Per-residue free energy decomposition (∆G_MM−GBSA_) with the following Equation (4):

(4) $$\Delta G_{\mathrm{MM}-\mathrm{GBSA}}=\Delta E_{\mathrm{vdW}}+\Delta E_{\mathrm{ele}}+\Delta E_{p}+\Delta E_{\mathrm{np}}$$

In this formula, ∆E_vdW_ represents the van der Waals interaction energy, ∆E_ele_ represents the electrostatic interaction energy, ∆E_p_ represents the polar solvation free energy and ∆E_np_ represents the nonpolar solvation free energy.

## 4. Conclusions

**Figure 13:**
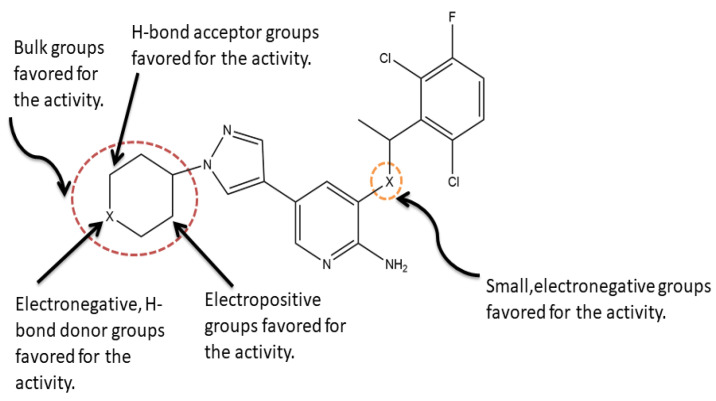
Graphical representation of SARs derived from CoMFA and CoMSIA analysis. All the groups mentioned above are beneficial to the inhibition activity.

In this study, a series of 2-aminopyridine inhibitors targeting c-Met kinase were used for molecular modeling (3D-QSAR, molecular docking and molecular dynamics simulations). The binding modes of the most active compound **4** showed that Arg1208 and Tyr1230 might be the key residues. 3D-QSAR including CoMFA and CoMSIA models were used to explore the relationships between 2-aminopyridine derivatives and their bioactivities. Especially the 3D contour maps can provide detailed understanding of the key structural features responsible for the c-Met kinase inhibitors activity, as summarized in Figure 13. The Results show that the electrostatic and hydrogen bonds are the main interactions between c-Met kinase and ligands, concordance with the docking result. The MD simulations were performed to study the system of the most active compound **4** bound to c-Met (3CCN). In this process, residue ASP1222 that formed hydrogen bonds with small molecules had the highest energy contribution to the integration of the entire system. The results from this study provide a reference for further rational design of novel c-Met kinase inhibitors with high potency.

## Figures and Tables

**Table 1:** The studied chemical IDs (the detailed molecular structures were listed in the Supplementary Materials) and corresponding experimental/predicted inhibition activities.

No.	Activity (pIC_50_)				
	Exp.	Alignment A		Alignment B	
		CoMFA	CoMSIA	CoMFA	CoMSIA
1	6.494	6.684	6.634	6.447	6.437
2	7.004	6.848	6.958	6.966	6.97
3	6.553	6.338	6.464	6.669	6.587
4	7.785	7.567	7.687	7.789	7.775
5	7.670	7.766	7.584	7.615	7.764
6	6.993	6.988	7.108	6.914	6.987
7	7.210	7.267	7.155	7.218	7.248
8	6.640	6.597	6.632	6.726	6.687
9	7.347	7.440	7.474	7.367	7.349
10 *	7.231	7.332	7.333	7.248	7.416
11 *	6.641	6.459	6.636	6.791	6.918
12 *	7.419	7.457	7.310	7.223	6.934
13	6.819	6.718	6.790	6.663	6.718
14	7.577	7.541	7.547	7.569	7.579
15	7.179	7.432	7.372	7.116	7.11
16*	6.885	6.841	6.917	6.884	6.909
17	7.297	7.284	7.288	7.333	7.223
18	7.588	7.324	7.259	7.553	7.6
19	7.343	7.426	7.301	7.425	7.373
20	7.348	7.358	7.332	7.365	7.362
21	6.619	6.745	7.018	6.64	6.577
22	7.115	7.019	7.084	7.096	7.031
23 *	7.088	7.021	7.145	6.87	7.024
24	7.656	7.647	7.685	7.789	7.67
25	6.996	6.892	6.786	6.97	7.026
26	7.188	7.193	7.239	7.158	7.283
27	7.009	7.070	7.000	6.965	6.968
28	6.707	6.791	6.750	6.707	6.655
29	6.761	6.771	6.784	6.747	6.755
30	5.920	5.766	5.797	5.833	5.736
31	5.155	5.450	5.600	5.354	5.46
32 *	6.222	6.237	6.356	6.369	6.263
33 *	5.386	6.036	6.075	5.984	5.505
34	6.319	6.040	5.980	6.366	6.347
35	5.886	5.821	5.833	5.75	5.762
36	5.943	6.084	6.026	6.063	6.009
37	6.387	6.375	6.269	6.435	6.491
38	6.251	6.223	6.193	6.171	6.13
39	6.292	6.356	6.178	6.304	6.357
40	6.678	6.843	6.892	6.683	6.681
41	5.943	6.006	5.969	5.9	5.963
42 *	6.468	6.887	6.863	6.102	6.202

* Prediction set compounds.

**Table 2:** Summary of the CoMFA and CoMSIA models for alignment-A and alignment-B.

PLS Statistic	Alignment-A		Alignment-B	
	CoMFA	CoMSIA	CoMFA	CoMSIA
Q^2^	0.596	0.646	0.563	0.568
ONC	2	2	6	2
R^2^	0.950	0.931	0.985	0.983
SEE	0.150	0.175	0.085	0.089
R^2^_pred_	0.839	0.840	0.821	0.854
F	160.303	76.047	286.459	260.420
Field distribution	-	-	-	-
S (Field distribution)	47.2%	19.7%	54.00%	14.30%
E (Field distribution)	52.8%	33.2%	46.00%	33.20%
A (Field distribution)	-	19.3%	-	30.00%
D (Field distribution)	-	27.8%	-	22.5%

**Table 3:** Different field combinations in CoMSIA analysis keeping S and E fields in all cases for alignment-A.

Field	ONC	Q^2^	R^2^	SEE	F	Rate of Contribution
SED	2	0.628	0.934	0.172	79.479	0.268:0.525:0.207
SEA	2	0.643	0.943	0.160	92.376	0.277:0.392:0.331
SEH	2	0.603	0.923	0.185	67.394	0.235:0.440:0.325
SEHA	2	0.620	0.929	0.179	73.004	0.191:0.292:0.255:0.262
SEHD	2	0.602	0.922	0.186	66.626	0.184:0.375:0.273:0.168
SEAD	2	0.646	0.931	0.175	76.047	0.197:0.332:0.193:0.278
SEHAD	2	0.622	0.922	0.187	66.342	0.148:0.253:0.160:0.227:0.212

**Table 4:** Different field combinations in CoMSIA analysis for alignment-B.

Field	ONC	Q^2^	R^2^	SEE	F	Rate of Contribution
SED	3	0.556	0.981	0.094	229.485	0.255:0.515:0.230
SEA	3	0.55	0.976	0.105	184.036	0.219:0.477:0.304
SEH	2	0.52	0.984	0.087	272.649	0.182:0.446:0.372
SEHA	2	0.53	0.985	0.082	303.786	0.136:0.339:0.306:0.219
SEHD	2	0.526	0.991	0.065	491.572	0.154:0.358:0.163:0.325
SEAD	3	0.561	0.981	0.095	228.254	0.178:0.378:0.264:0.180
SEHDA	2	0.536	0.988	0.073	385.686	0.117:0.286:0.197:0.138:0.262

**Table 5:** Binding free energy of the system and energy contribution of each component.

Interaction	Contribution (kcal/mol)	Standard Deviation
∆E_vdW_	−32.99	3.62
∆E_ele_	36.18	24.78
∆G_GB_	−25.82	23.74
∆G_SA_	−4.45	0.38
∆E_gas_	3.19	24.72
∆E_solv_	−30.27	23.64
∆G_bind_	−27.08	3.80

**Table 6:** Hydrogen bond distribution for the system.

Acceptor	DonorH	Donor	Frac
ASP_1222@OD2	MOL@H22	MOL@N4	0.6651
ASP_1222@OD2	MOL@H20	MOL@N3	0.6569
ASP_1222@OD1	MOL@H20	MOL@N3	0.6431
ASP_1222@OD1	MOL@H22	MOL@N4	0.6178

## Data Availability

The data presented in this study are available in the Supplementary Materials.

## Supplementary Materials

The following are available online. The SMILE strings and corresponding structures of all the compounds used in this study, as numbered in Table 1.

## Sample Availability

Samples of the compounds are not available from the authors.
