# Supplementary material for: Studying the Binding Modes of Novel 2-Aminopyridine Derivatives as Effective and Selective c-Met Kinase Type 1 Inhibitors Using Molecular Modeling Approaches
Source: Molecules. 2020 Dec 24;26(1):52. doi: 10.3390/molecules26010052 (PMC7795969; doi:10.3390/molecules26010052)

Supplementary Materials

The SMILE strings and corresponding structures of all the compounds used in this study, as numbered in Table 1.

1.NC(C(C(NC(C1=C(Cl)C=CC(F)=C1Cl)C)=O)=C2)=NC=C2C3=CN(C4CCNCC4)N=C3

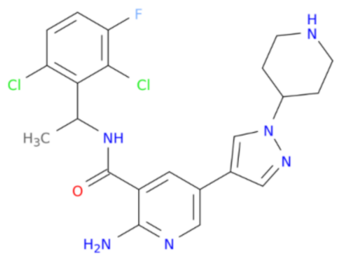

2.NC(C(C(NC(C1=C(Cl)C=CC(F)=C1Cl)C)=O)=C2)=NC=C2C3=CN(C4CCOCC4)N=C3

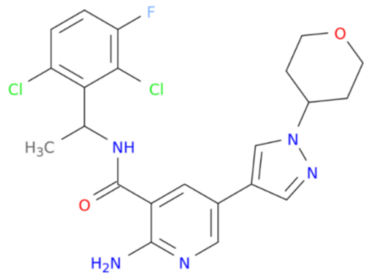

3.CN1C=C(C2=CN=C(N)C(C(NC(C3=C(Cl)C=CC(F)=C3Cl)C)=O)=C2)C=N1

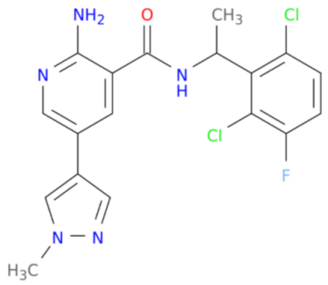

4.NC(C(SC(C1=C(Cl)C=CC(F)=C1Cl)C)=C2)=NC=C2C3=CN(C4CCNCC4)N=C3

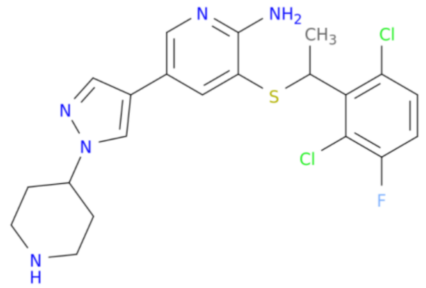

5.NC(C(SC(C1=C(Cl)C=CC(F)=C1Cl)C)=C2)=NC=C2C3=CN(C4CCOCC4)N=C3

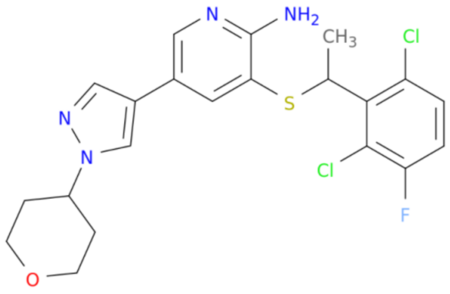

6.NC(C(SC(C1=C(Cl)C=CC(F)=C1Cl)C)=C2)=NC=C2C3=CN(C)N=C3

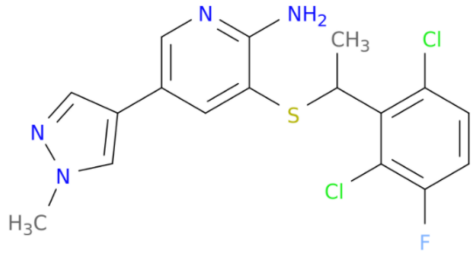

7.NC(C(SC(C1=C(Cl)C=CC(F)=C1Cl)C)=C2)=NC=C2C3=CN(CCO)N=C3

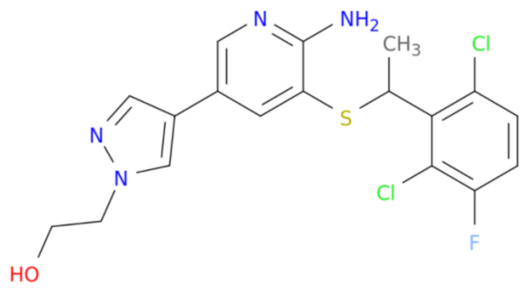

8.NC(C(SC(C1=C(Cl)C=CC(F)=C1Cl)C)=C2)=NC=C2C3=CN(C(N(C)C)=O)N=C3

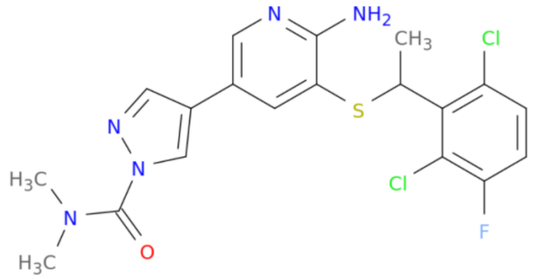

9.NC(C(SC(C1=C(Cl)C=CC(F)=C1Cl)C)=C2)=NC=C2C3=CN(CC(N(C)C)=O)N=C3

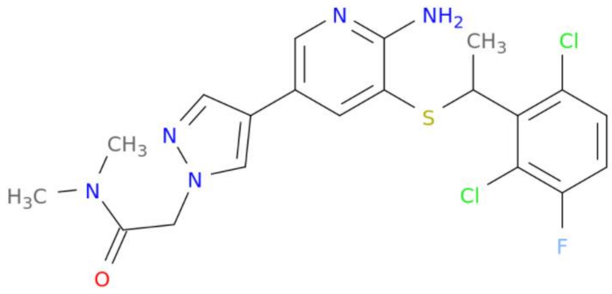

10.NC(C(SC(C1=C(Cl)C=CC(F)=C1Cl)C)=C2)=NC=C2C3=CN(C4CCN(S(C)(=O)=O)CC4)N=C3

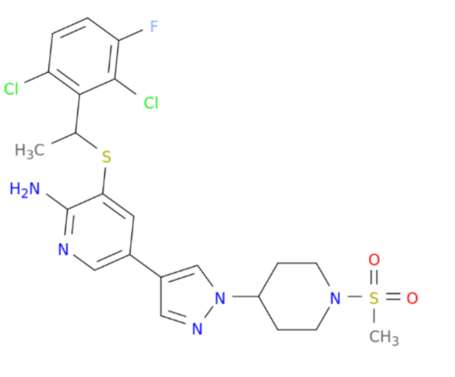

11.NC(C(SC(C1=C(Cl)C=CC(F)=C1Cl)C)=C2)=NC=C2C3=CN(C4CCN(C(N(C)C)=O)CC4)N=C3

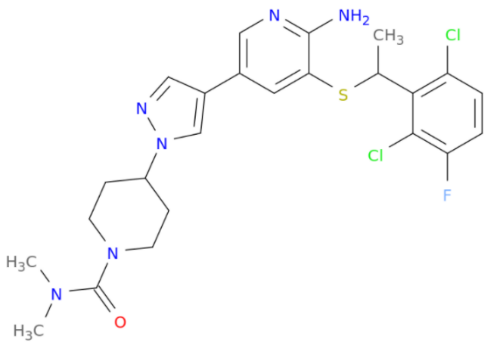

12.NC(C(SC(C1=C(Cl)C=CC(F)=C1Cl)C)=C2)=NC=C2C3=CN(C4CCN(C(CO)=O)CC4)N=C3

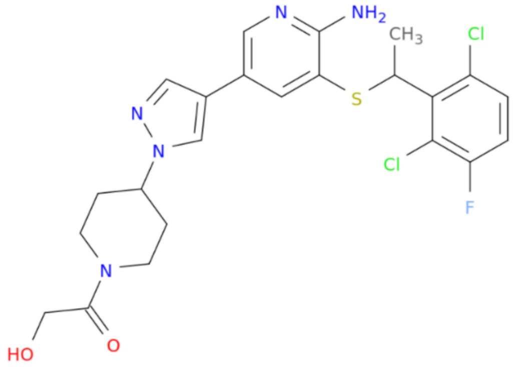

13.NC1=NC=C(C2=CN=CN=C2)C=C1SC(C3=C(Cl)C=CC(F)=C3Cl)C

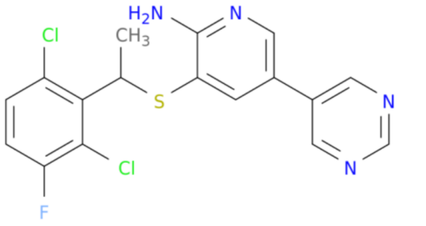

14.NC1=NC=C(C2=CC=C(N3CCN(C)CC3)N=C2)C=C1SC(C4=C(Cl)C=CC(F)=C4Cl)C

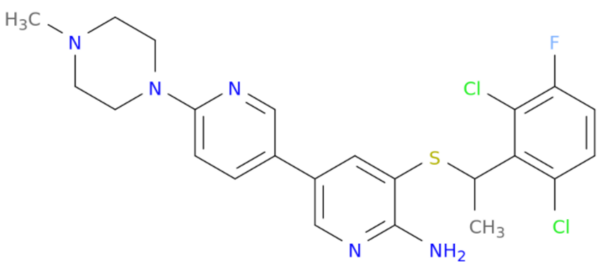

15.NC1=NC=C(C2=CC=C(N3CCOCC3)N=C2)C=C1SC(C4=C(Cl)C=CC(F)=C4Cl)C

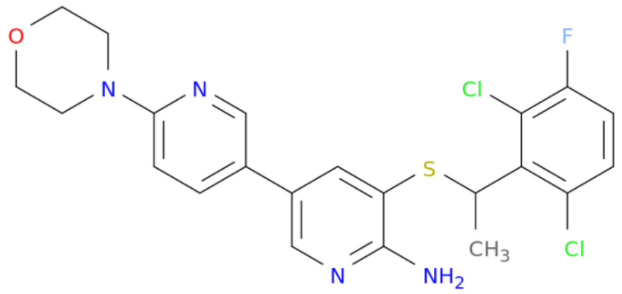

16.NC1=NC=C(C2=CC3=C(NC(C3)=O)C=C2)C=C1SC(C4=C(Cl)C=CC(F)=C4Cl)C

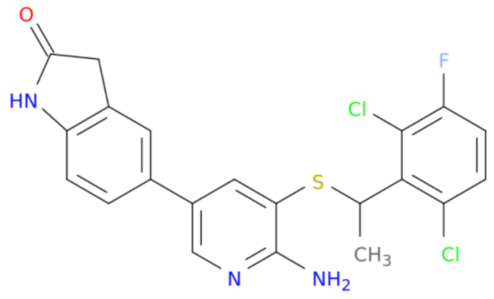

17.NC1=NC=C(C2=CC=C(OCC(N3CCOCC3)=O)C=C2)C=C1SC(C4=C(Cl)C=CC(F)=C4Cl)C

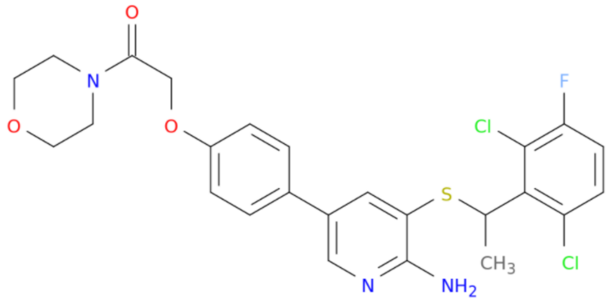

18.NC1=NC=C(C2=CC=C(C(N3CCN(C)CC3)=O)C=C2)C=C1SC(C4=C(Cl)C=CC(F)=C4Cl)C

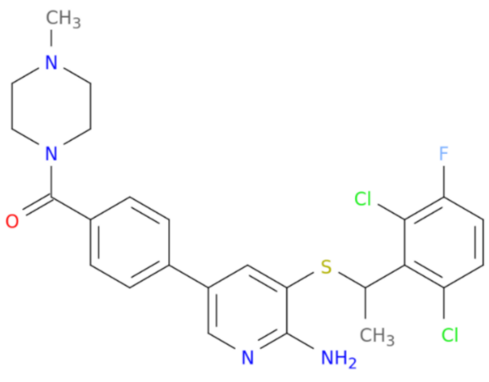

19.NC1=NC=C(C2=CC=C(C(N3CCOCC3)=O)C=C2)C=C1SC(C4=C(Cl)C=CC(F)=C4Cl)C

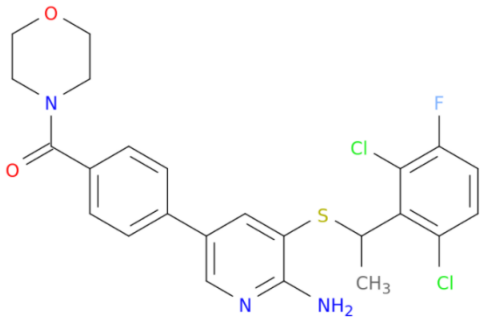

20.NC1=NC=C(C2=CC=C(C(N(C)C)=O)C=C2)C=C1SC(C3=C(Cl)C=CC(F)=C3Cl)C

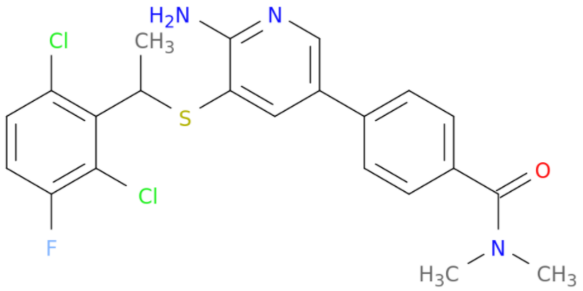

21.NC1=NC=C(C2=CC=C(C(N3CCN(C(C)C)CC3)=O)C=C2)C=C1SC(C4=C(Cl)C=CC(F)=C4Cl)C

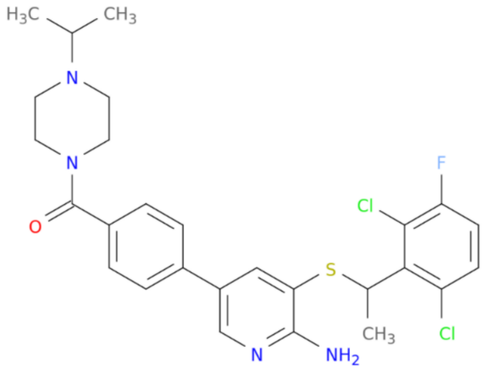

22.NC1=NC=C(C2=CC=C(C(N3CCN(S(=O)(C)=O)CC3)=O)C=C2)C=C1SC(C4=C(Cl)C=CC(F)=C4Cl)C

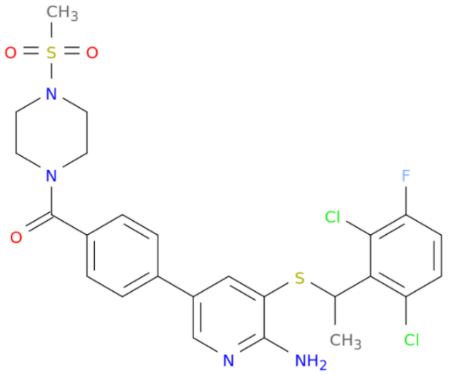

23.NC1=NC=C(C2=CC=C(C(N3CCN(CCO)CC3)=O)C=C2)C=C1SC(C4=C(Cl)C=CC(F)=C4Cl)C

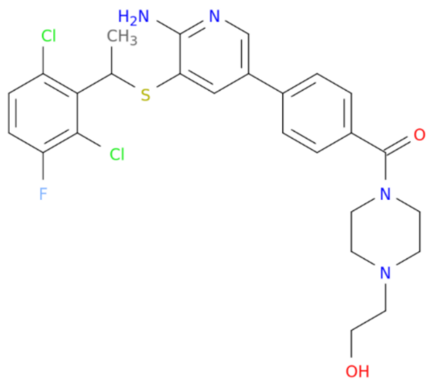

24.NC1=NC=C(C2=CC=C(C(N3CCC(N4CCCC4)CC3)=O)C=C2)C=C1SC(C5=C(Cl)C=CC(F)=C5Cl)C

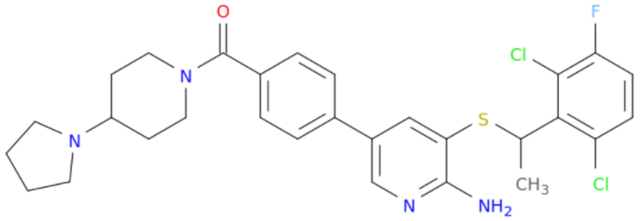

25.NC1=NC=C(C2=CC=C(C(NCCN(CC)CC)=O)C=C2)C=C1SC(C3=C(Cl)C=CC(F)=C3Cl)C

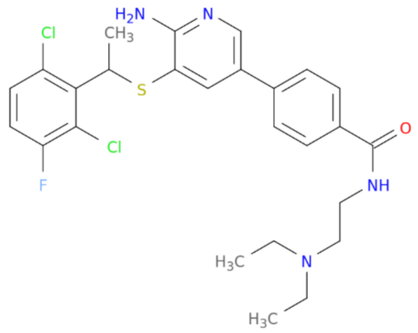

26.NC1=NC=C(C2=CC=C(C(N3CCN(C)CC3)=O)C(F)=C2)C=C1SC(C4=C(Cl)C=CC(F)=C4Cl)C

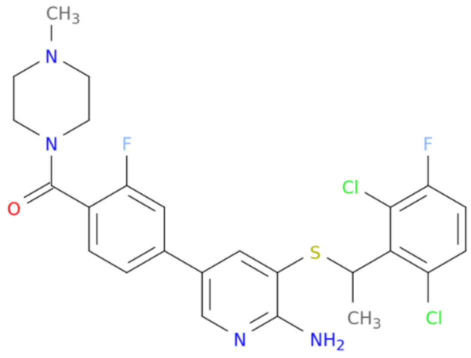

27.NC1=NC=C(C2=CC=C(C(N3CCOCC3)=O)C(F)=C2)C=C1SC(C4=C(Cl)C=CC(F)=C4Cl)C

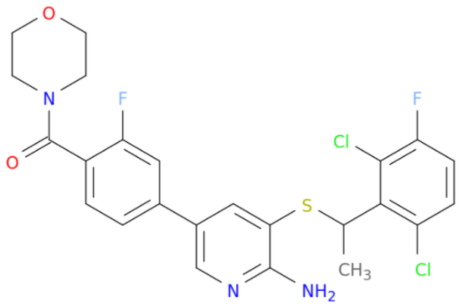

28.NC1=NC=C(C2=CC=C(C(N(C)C)=O)C(F)=C2)C=C1SC(C3=C(Cl)C=CC(F)=C3Cl)C

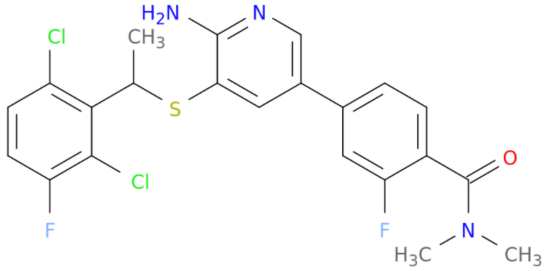

29.NC1=NC=C(C2=CC=C(C(N3CCC(N4CCCC4)CC3)=O)C(F)=C2)C=C1SC(C5=C(Cl)C=CC(F)=C5Cl)C

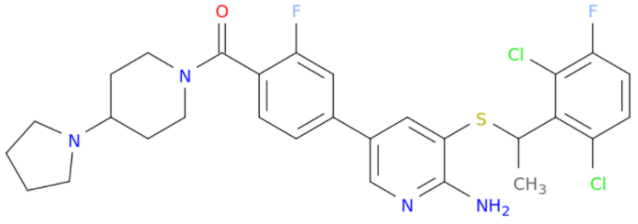

30.NC1=NC=C(C2=CN(C)N=C2)C=C1C(CCC3=C(F)C(F)=CC=C3)=O

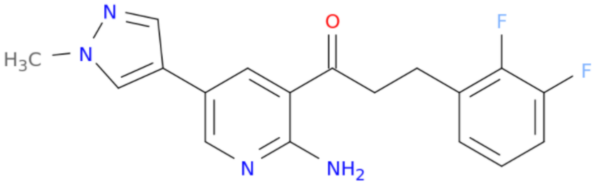

31.NC1=NC=C(C2=CN(C)N=C2)C=C1C(CCC3=C(F)C=C(F)C=C3)=O

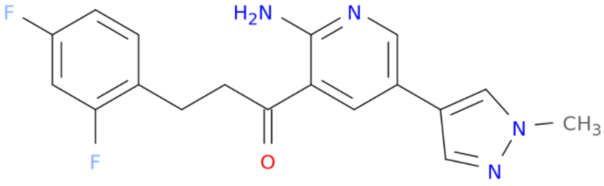

32.NC1=NC=C(C2=CN(C)N=C2)C=C1C(CCC3=C(F)C=CC(F)=C3)=O

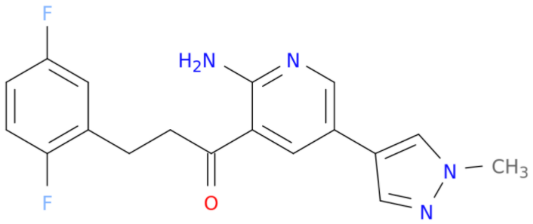

33.NC1=NC=C(C2=CN(C)N=C2)C=C1C(CCC3=CC(F)=CC(F)=C3)=O

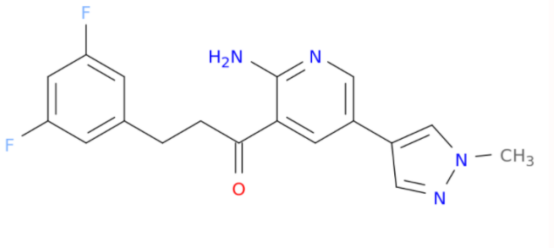

34.NC1=NC=C(C2=CN(C)N=C2)C=C1C(CCC3=CC(F)=C(F)C=C3)=O

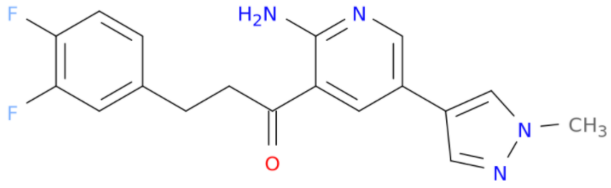

35.NC1=NC=C(C2=CN(C)N=C2)C=C1C(CCC3=C(F)C=CC=C3Cl)=O

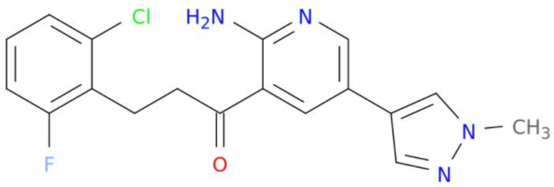

36.NC1=NC=C(C2=CN(C)N=C2)C=C1C(CCC3=C(F)C(Cl)=CC=C3F)=O

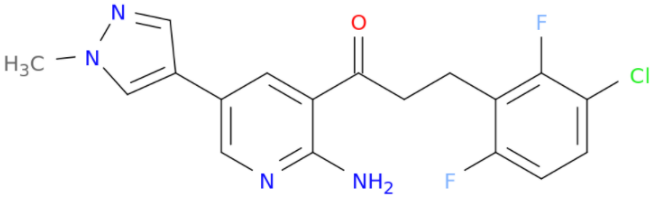

37.NC1=NC=C(C2=CN(C)N=C2)C=C1C(CCC3=C(Cl)C(F)=CC=C3F)=O

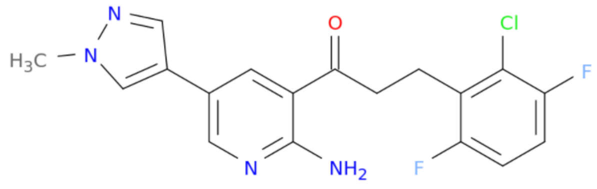

38.NC1=NC=C(C2=CN(C)N=C2)C=C1C(CCC3=C(Cl)C(Cl)=CC=C3F)=O

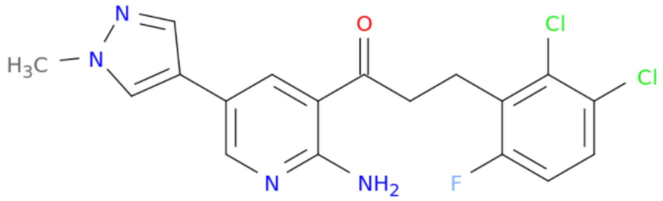

39.NC1=NC=C(C2=CN=C(N3CCOCC3)C=C2)C=C1C(NC(C)C4=C(Cl)C=CC(F)=C4Cl)=O

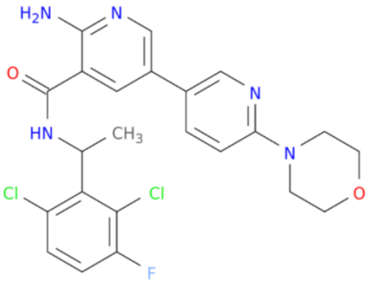

40.NC1=NC=C(C2=CC3=C(NC(C3)=O)C=C2)C=C1C(NC(C)C4=C(Cl)C=CC(F)=C4Cl)=O

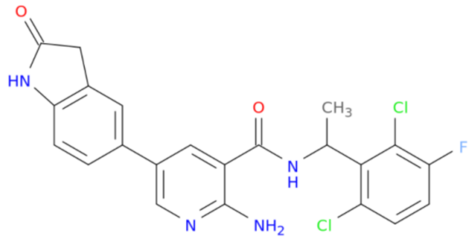

41.NC1=NC=C(C2=CN=CN=C2)C=C1C(NC(C)C3=C(Cl)C=CC(F)=C3Cl)=O

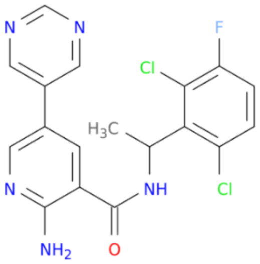

42.NC1=NC=C(C2=CC=C(OCC(N3CCOCC3)=O)C=C2)C=C1C(NC(C)C4=C(Cl)C=CC(F)=C4Cl)=O

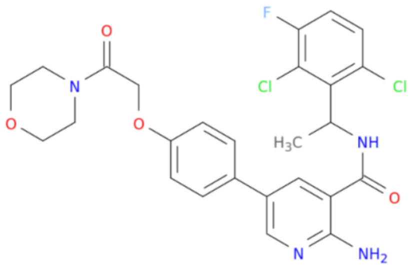

Supplement: Supplementary file 1 [file molecules-26-00052-s001.pdf]
